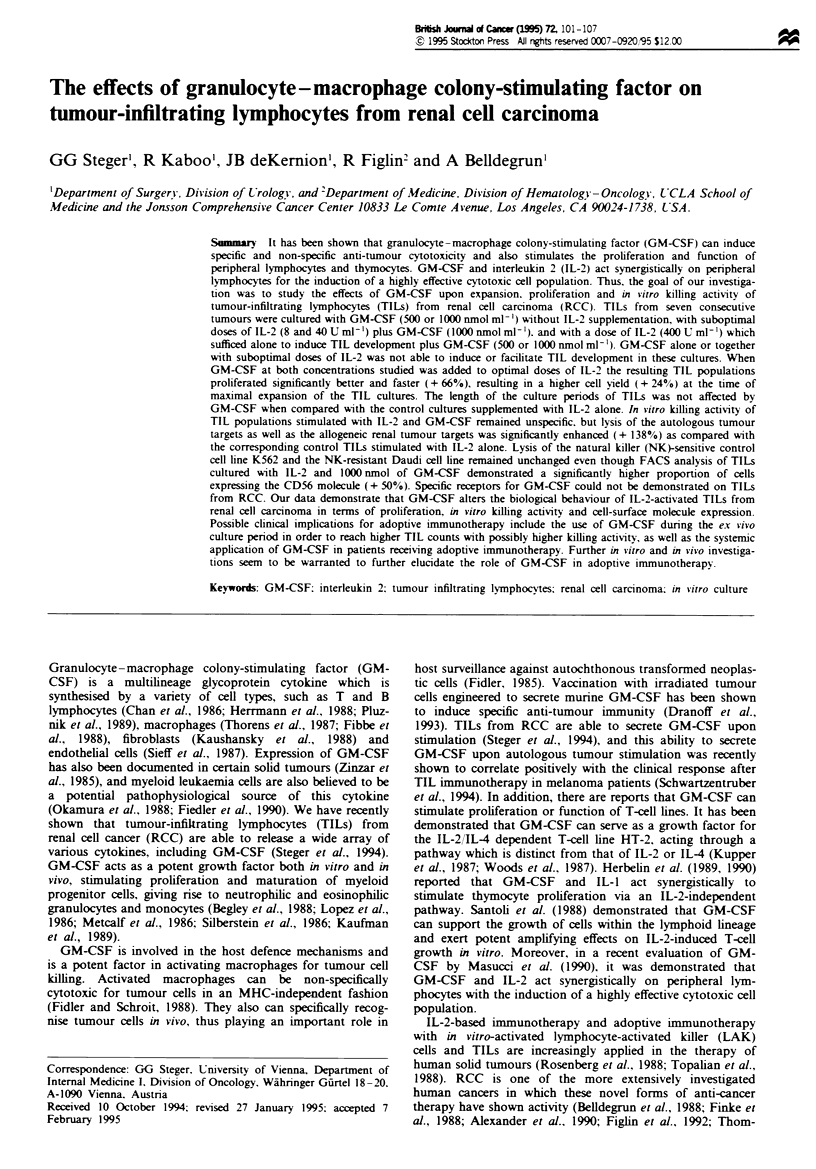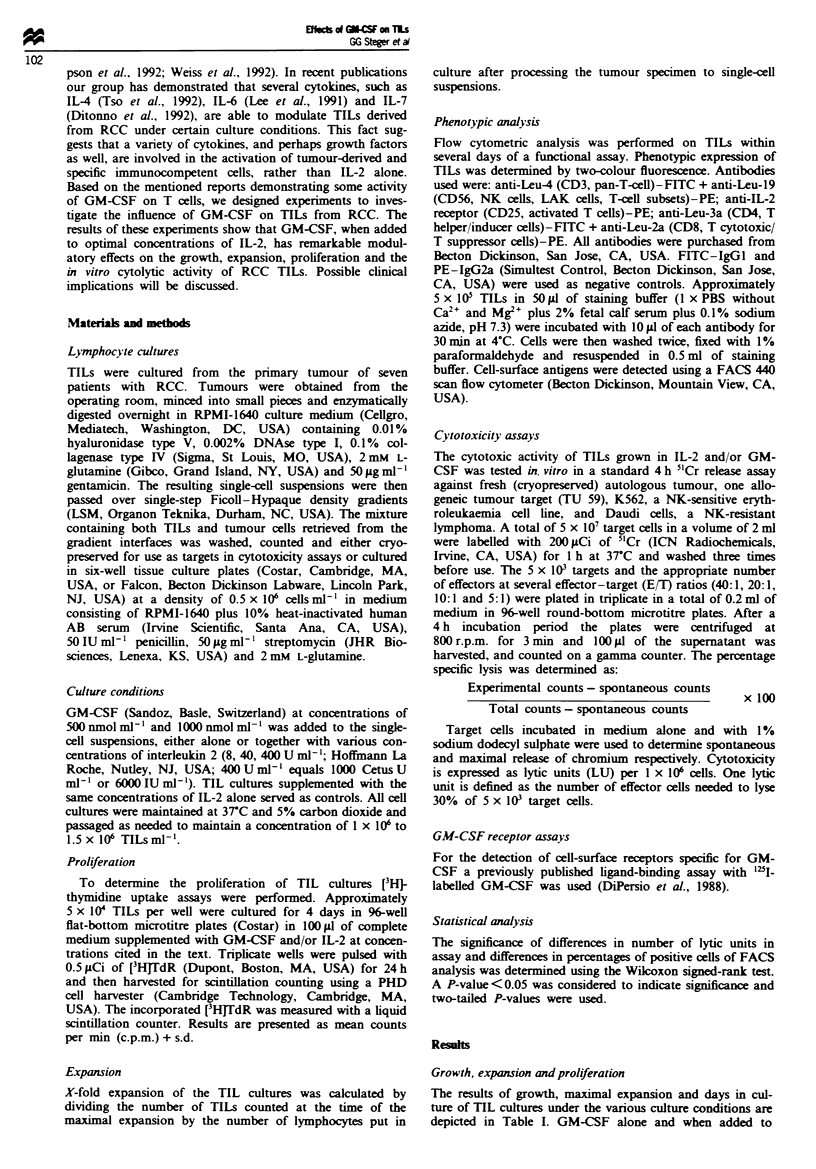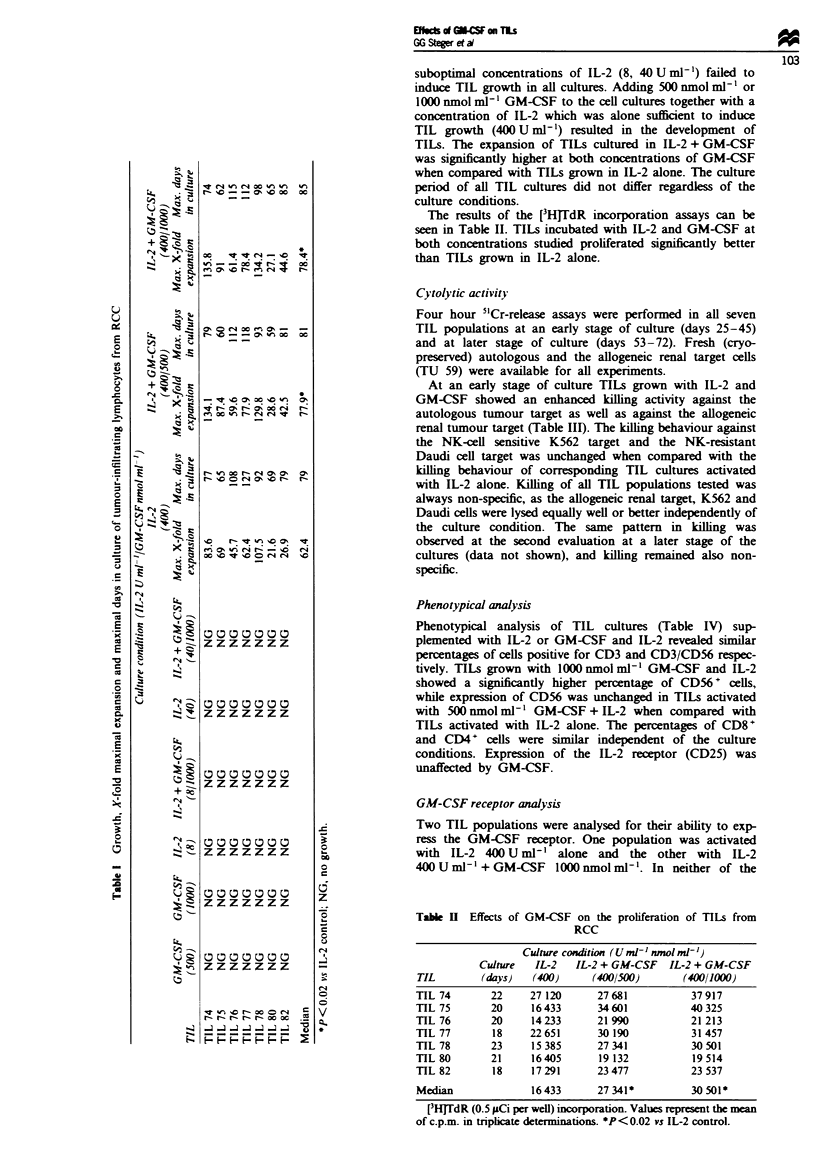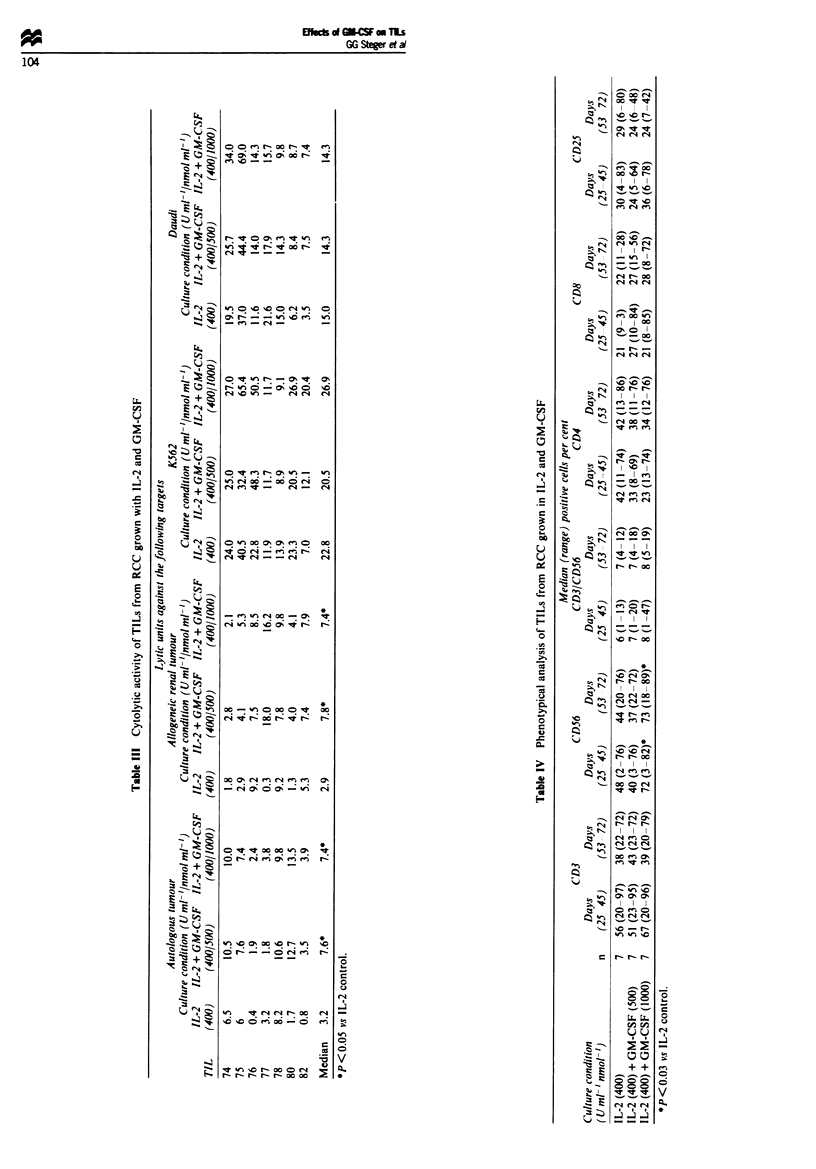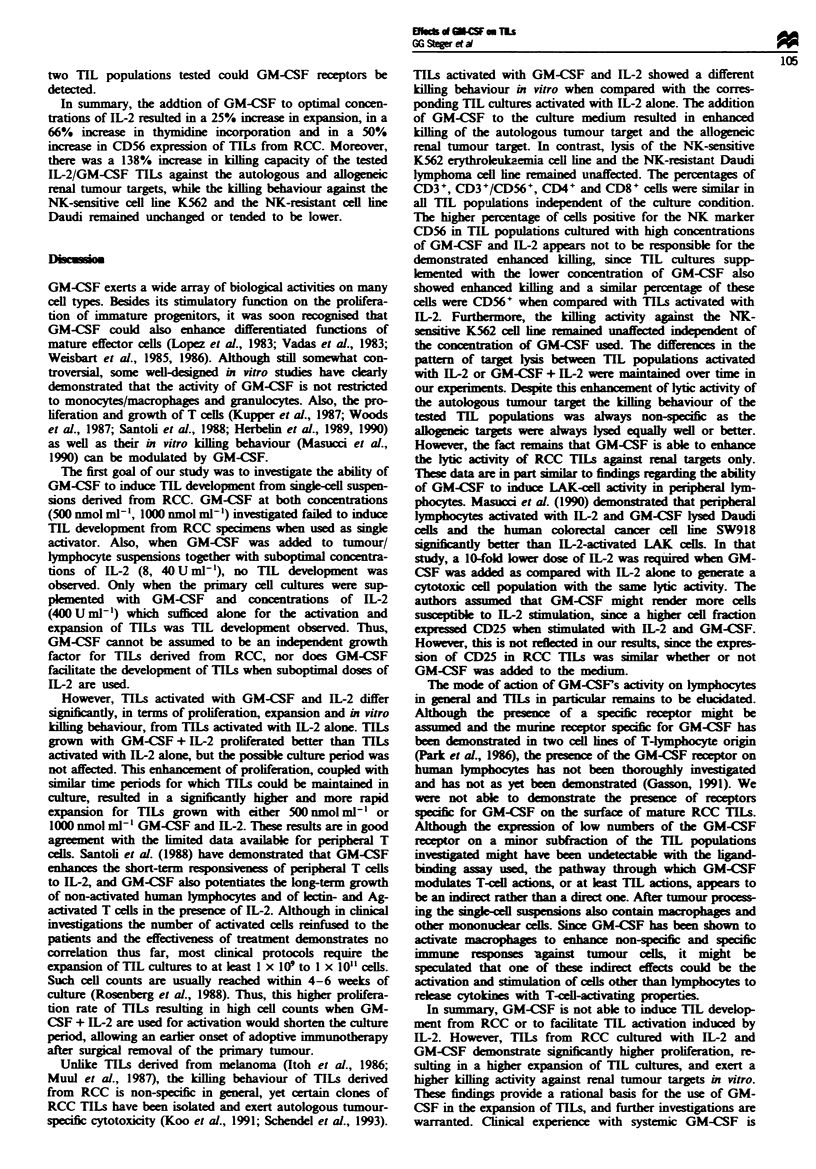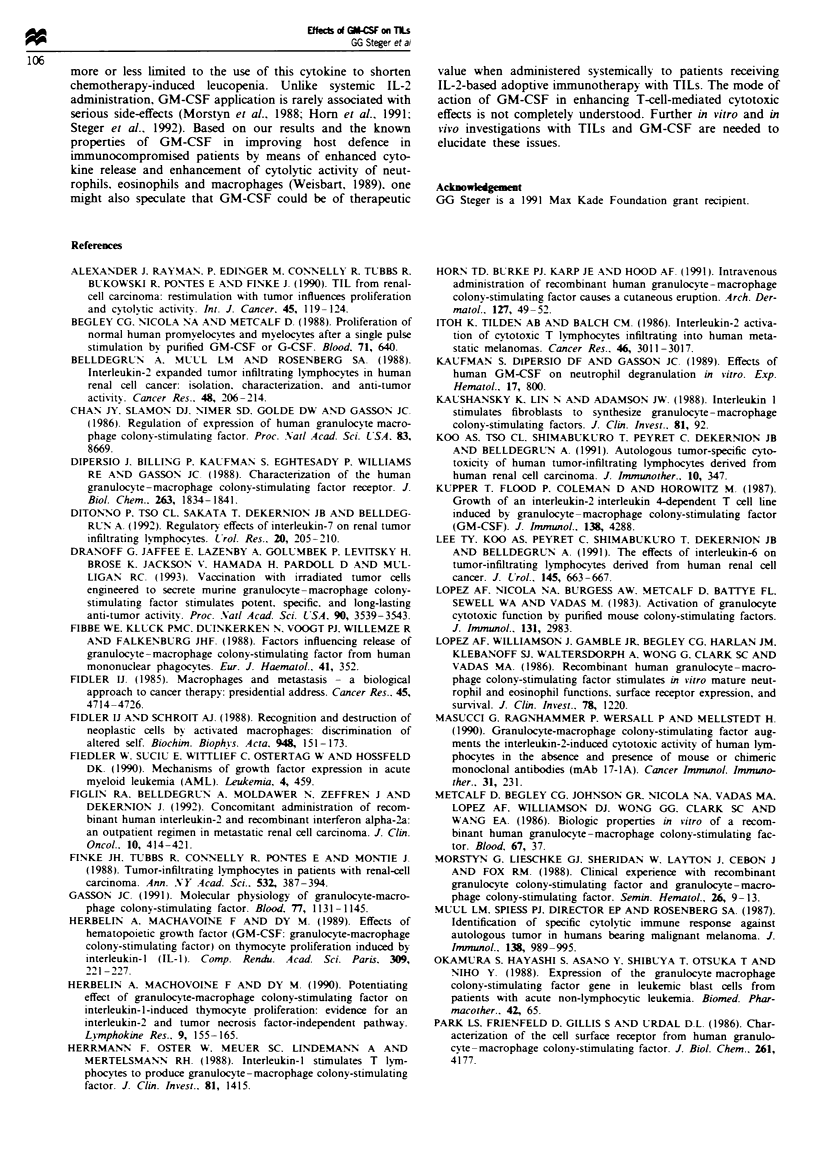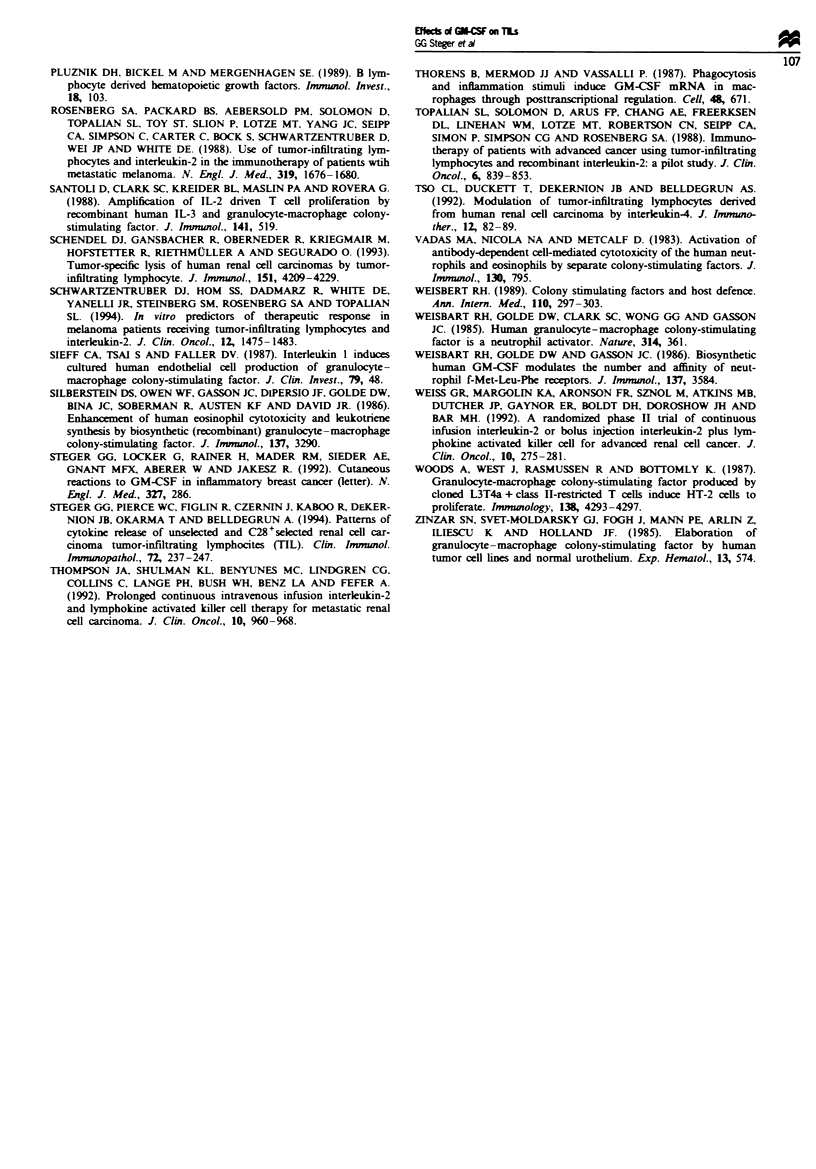# The effects of granulocyte-macrophage colony-stimulating factor on tumour-infiltrating lymphocytes from renal cell carcinoma.

**DOI:** 10.1038/bjc.1995.284

**Published:** 1995-07

**Authors:** G. G. Steger, R. Kaboo, J. B. deKernion, R. Figlin, A. Belldegrun

**Affiliations:** Department of Surgery, UCLA School of Medicine 90024-1738, USA.

## Abstract

It has been shown that granulocyte-macrophage colony-stimulating factor (GM-CSF) can induce specific and non-specific anti-tumour cytotoxicity and also stimulates the proliferation and function of peripheral lymphocytes and thymocytes. GM-CSF and interleukin 2 (IL-2) act synergistically on peripheral lymphocytes for the induction of a highly effective cytotoxic cell population. Thus, the goal of our investigation was to study the effects of GM-CSF upon expansion, proliferation and in vitro killing activity of tumour-infiltrating lymphocytes (TILs) from renal cell carcinoma (RCC). TILs from seven consecutive tumours were cultured with GM-CSF (500 or 1000 nmol ml-1) without IL-2 supplementation, with suboptimal doses of IL-2 (8 and 40 U ml-1) plus GM-CSF (1000 nmol ml-1), and with a dose of IL-2 (400 U ml-1) which sufficed alone to induce TIL development plus GM-CSF (500 or 1000 nmol ml-1). GM-CSF alone or together with suboptimal doses of IL-2 was not able to induce or facilitate TIL development in these cultures. When GM-CSF at both concentrations studied was added to optimal doses of IL-2 the resulting TIL populations proliferated significantly better and faster (+66%), resulting in a higher cell yield (+24%) at the time of maximal expansion of the TIL cultures. The length of the culture periods of TILs was not affected by GM-CSF when compared with the control cultures supplemented with IL-2 alone. In vitro killing activity of TIL populations stimulated with IL-2 and GM-CSF remained unspecific, but lysis of the autologous tumour targets as well as the allogeneic renal tumour targets was significantly enhanced (+138%) as compared with the corresponding control TILs stimulated with IL-2 alone. Lysis of the natural killer (NK)-sensitive control cell line K562 and the NK-resistant Daudi cell line remained unchanged even though FACS analysis of TILs cultured with IL-2 and 1000 nmol of GM-CSF demonstrated a significantly higher proportion of cells expressing the CD56 molecule (+50%). Specific receptors for GM-CSF could not be demonstrated on TILs from RCC. Our data demonstrate that GM-CSF alters the biological behaviour of IL-2-activated TILs from renal cell carcinoma in terms of proliferation, in vitro killing activity and cell-surface molecule expression.(ABSTRACT TRUNCATED AT 400 WORDS)


© 1995 Stockton Press All rights reserved 0007-0920/95 $12.00

### The effects of granulocyte-macrophage colony-stimulating factor on tumour-infiltrating lymphocytes from renal cell carcinoma

GG Steger[1], R Kaboo[1], JB deKernion[1], R Figlin[2] and A Belldegrun[1]

[1]*Department of Surgery, Division of Urology, and* [2]*Department of Medicine, Division of Hematology-Oncology, UCLA School of Medicine and the Jonsson Comprehensive Cancer Center 10833 Le Comte Avenue, Los Angeles, CA 90024-1738, USA.*

**Summary** It has been shown that granulocyte-macrophage colony-stimulating factor (GM-CSF) can induce specific and non-specific anti-tumour cytotoxicity and also stimulates the proliferation and function of peripheral lymphocytes and thymocytes. GM-CSF and interleukin 2 (IL-2) act synergistically on peripheral lymphocytes for the induction of a highly effective cytotoxic cell population. Thus, the goal of our investigation was to study the effects of GM-CSF upon expansion, proliferation and *in vitro* killing activity of tumour-infiltrating lymphocytes (TILs) from renal cell carcinoma (RCC). TILs from seven consecutive tumours were cultured with GM-CSF (500 or 1000 nmol $ml^{-1}$) without IL-2 supplementation, with suboptimal doses of IL-2 (8 and 40 U $ml^{-1}$) plus GM-CSF (1000 nmol $ml^{-1}$), and with a dose of IL-2 (400 U $ml^{-1}$) which sufficed alone to induce TIL development plus GM-CSF (500 or 1000 nmol $ml^{-1}$). GM-CSF alone or together with suboptimal doses of IL-2 was not able to induce or facilitate TIL development in these cultures. When GM-CSF at both concentrations studied was added to optimal doses of IL-2 the resulting TIL populations proliferated significantly better and faster (+66%), resulting in a higher cell yield (+24%) at the time of maximal expansion of the TIL cultures. The length of the culture periods of TILs was not affected by GM-CSF when compared with the control cultures supplemented with IL-2 alone. *In vitro* killing activity of TIL populations stimulated with IL-2 and GM-CSF remained unspecific, but lysis of the autologous tumour targets as well as the allogeneic renal tumour targets was significantly enhanced (+138%) as compared with the corresponding control TILs stimulated with IL-2 alone. Lysis of the natural killer (NK)-sensitive control cell line K562 and the NK-resistant Daudi cell line remained unchanged even though FACS analysis of TILs cultured with IL-2 and 1000 nmol of GM-CSF demonstrated a significantly higher proportion of cells expressing the CD56 molecule (+50%). Specific receptors for GM-CSF could not be demonstrated on TILs from RCC. Our data demonstrate that GM-CSF alters the biological behaviour of IL-2-activated TILs from renal cell carcinoma in terms of proliferation, *in vitro* killing activity and cell-surface molecule expression. Possible clinical implications for adoptive immunotherapy include the use of GM-CSF during the *ex vivo* culture period in order to reach higher TIL counts with possibly higher killing activity, as well as the systemic application of GM-CSF in patients receiving adoptive immunotherapy. Further *in vitro* and *in vivo* investigations seem to be warranted to further elucidate the role of GM-CSF in adoptive immunotherapy.

**Keywords**: GM-CSF; interleukin 2; tumour infiltrating lymphocytes; renal cell carcinoma; *in vitro* culture

Granulocyte-macrophage colony-stimulating factor (GM-CSF) is a multilineage glycoprotein cytokine which is synthesised by a variety of cell types, such as T and B lymphocytes (Chan *et al.*, 1986; Herrmann *et al.*, 1988; Pluznik *et al.*, 1989), macrophages (Thorens *et al.*, 1987; Fibbe *et al.*, 1988), fibroblasts (Kaushansky *et al.*, 1988) and endothelial cells (Sieff *et al.*, 1987). Expression of GM-CSF has also been documented in certain solid tumours (Zinzar *et al.*, 1985), and myeloid leukaemia cells are also believed to be a potential pathophysiological source of this cytokine (Okamura *et al.*, 1988; Fiedler *et al.*, 1990). We have recently shown that tumour-infiltrating lymphocytes (TILs) from renal cell cancer (RCC) are able to release a wide array of various cytokines, including GM-CSF (Steger *et al.*, 1994). GM-CSF acts as a potent growth factor both *in vitro* and *in vivo*, stimulating proliferation and maturation of myeloid progenitor cells, giving rise to neutrophilic and eosinophilic granulocytes and monocytes (Begley *et al.*, 1988; Lopez *et al.*, 1986; Metcalf *et al.*, 1986; Silberstein *et al.*, 1986; Kaufman *et al.*, 1989).

GM-CSF is involved in the host defence mechanisms and is a potent factor in activating macrophages for tumour cell killing. Activated macrophages can be non-specifically cytotoxic for tumour cells in an MHC-independent fashion (Fidler and Schroit, 1988). They also can specifically recognise tumour cells *in vivo*, thus playing an important role in host surveillance against autochthonous transformed neoplastic cells (Fidler, 1985). Vaccination with irradiated tumour cells engineered to secrete murine GM-CSF has been shown to induce specific anti-tumour immunity (Dranoff *et al.*, 1993). TILs from RCC are able to secrete GM-CSF upon stimulation (Steger *et al.*, 1994), and this ability to secrete GM-CSF upon autologous tumour stimulation was recently shown to correlate positively with the clinical response after TIL immunotherapy in melanoma patients (Schwartzentruber *et al.*, 1994). In addition, there are reports that GM-CSF can stimulate proliferation or function of T-cell lines. It has been demonstrated that GM-CSF can serve as a growth factor for the IL-2/IL-4 dependent T-cell line HT-2, acting through a pathway which is distinct from that of IL-2 or IL-4 (Kupper *et al.*, 1987; Woods *et al.*, 1987). Herbelin *et al.* (1989, 1990) reported that GM-CSF and IL-1 act synergistically to stimulate thymocyte proliferation via an IL-2-independent pathway. Santoli *et al.* (1988) demonstrated that GM-CSF can support the growth of cells within the lymphoid lineage and exert potent amplifying effects on IL-2-induced T-cell growth *in vitro*. Moreover, in a recent evaluation of GM-CSF by Masucci *et al.* (1990), it was demonstrated that GM-CSF and IL-2 act synergistically on peripheral lymphocytes with the induction of a highly effective cytotoxic cell population.

IL-2-based immunotherapy and adoptive immunotherapy with *in vitro*-activated lymphocyte-activated killer (LAK) cells and TILs are increasingly applied in the therapy of human solid tumours (Rosenberg *et al.*, 1988; Topalian *et al.*, 1988). RCC is one of the more extensively investigated human cancers in which these novel forms of anti-cancer therapy have shown activity (Belldegrun *et al.*, 1988; Finke *et al.*, 1988; Alexander *et al.*, 1990; Figlin *et al.*, 1992; Thom-

Correspondence: GG Steger, University of Vienna, Department of Internal Medicine I, Division of Oncology, Währinger Gürtel 18-20, A-1090 Vienna, Austria
Received 10 October 1994; revised 27 January 1995; accepted 7 February 1995

pson et al., 1992; Weiss et al., 1992). In recent publications our group has demonstrated that several cytokines, such as IL-4 (Tso et al., 1992), IL-6 (Lee et al., 1991) and IL-7 (Ditonno et al., 1992), are able to modulate TILs derived from RCC under certain culture conditions. This fact suggests that a variety of cytokines, and perhaps growth factors as well, are involved in the activation of tumour-derived and specific immunocompetent cells, rather than IL-2 alone. Based on the mentioned reports demonstrating some activity of GM-CSF on T cells, we designed experiments to investigate the influence of GM-CSF on TILs from RCC. The results of these experiments show that GM-CSF, when added to optimal concentrations of IL-2, has remarkable modulatory effects on the growth, expansion, proliferation and the *in vitro* cytolytic activity of RCC TILs. Possible clinical implications will be discussed.

### Materials and methods

### *Lymphocyte cultures*

TILs were cultured from the primary tumour of seven patients with RCC. Tumours were obtained from the operating room, minced into small pieces and enzymatically digested overnight in RPMI-1640 culture medium (Cellgro, Mediatech, Washington, DC, USA) containing 0.01% hyaluronidase type V, 0.002% DNAse type I, 0.1% collagenase type IV (Sigma, St Louis, MO, USA), 2 mM L-glutamine (Gibco, Grand Island, NY, USA) and 50 $\mu g\ ml^{-1}$ gentamicin. The resulting single-cell suspensions were then passed over single-step Ficoll–Hypaque density gradients (LSM, Organon Teknika, Durham, NC, USA). The mixture containing both TILs and tumour cells retrieved from the gradient interfaces was washed, counted and either cryopreserved for use as targets in cytotoxicity assays or cultured in six-well tissue culture plates (Costar, Cambridge, MA, USA, or Falcon, Becton Dickinson Labware, Lincoln Park, NJ, USA) at a density of $0.5 \times 10^6$ cells $ml^{-1}$ in medium consisting of RPMI-1640 plus 10% heat-inactivated human AB serum (Irvine Scientific, Santa Ana, CA, USA), 50 IU $ml^{-1}$ penicillin, 50 $\mu g\ ml^{-1}$ streptomycin (JHR Biosciences, Lenexa, KS, USA) and 2 mM L-glutamine.

### *Culture conditions*

GM-CSF (Sandoz, Basle, Switzerland) at concentrations of 500 nmol $ml^{-1}$ and 1000 nmol $ml^{-1}$ was added to the single-cell suspensions, either alone or together with various concentrations of interleukin 2 (8, 40, 400 U $ml^{-1}$; Hoffmann La Roche, Nutley, NJ, USA; 400 U $ml^{-1}$ equals 1000 Cetus U $ml^{-1}$ or 6000 IU $ml^{-1}$). TIL cultures supplemented with the same concentrations of IL-2 alone served as controls. All cell cultures were maintained at 37°C and 5% carbon dioxide and passaged as needed to maintain a concentration of $1 \times 10^6$ to $1.5 \times 10^6$ TILs $ml^{-1}$.

### *Proliferation*

To determine the proliferation of TIL cultures [$^3$H]-thymidine uptake assays were performed. Approximately $5 \times 10^4$ TILs per well were cultured for 4 days in 96-well flat-bottom microtitre plates (Costar) in 100 µl of complete medium supplemented with GM-CSF and/or IL-2 at concentrations cited in the text. Triplicate wells were pulsed with 0.5 µCi of [$^3$H]TdR (Dupont, Boston, MA, USA) for 24 h and then harvested for scintillation counting using a PHD cell harvester (Cambridge Technology, Cambridge, MA, USA). The incorporated [$^3$H]TdR was measured with a liquid scintillation counter. Results are presented as mean counts per min (c.p.m.) + s.d.

### *Expansion*

*X*-fold expansion of the TIL cultures was calculated by dividing the number of TILs counted at the time of the maximal expansion by the number of lymphocytes put in culture after processing the tumour specimen to single-cell suspensions.

### *Phenotypic analysis*

Flow cytometric analysis was performed on TILs within several days of a functional assay. Phenotypic expression of TILs was determined by two-colour fluorescence. Antibodies used were: anti-Leu-4 (CD3, pan-T-cell)–FITC + anti-Leu-19 (CD56, NK cells, LAK cells, T-cell subsets)–PE; anti-IL-2 receptor (CD25, activated T cells)–PE; anti-Leu-3a (CD4, T helper/inducer cells)–FITC + anti-Leu-2a (CD8, T cytotoxic/T suppressor cells)–PE. All antibodies were purchased from Becton Dickinson, San Jose, CA, USA. FITC–IgG1 and PE–IgG2a (Simultest Control, Becton Dickinson, San Jose, CA, USA) were used as negative controls. Approximately $5 \times 10^5$ TILs in 50 µl of staining buffer (1 × PBS without $Ca^{2+}$ and $Mg^{2+}$ plus 2% fetal calf serum plus 0.1% sodium azide, pH 7.3) were incubated with 10 µl of each antibody for 30 min at 4°C. Cells were then washed twice, fixed with 1% paraformaldehyde and resuspended in 0.5 ml of staining buffer. Cell-surface antigens were detected using a FACS 440 scan flow cytometer (Becton Dickinson, Mountain View, CA, USA).

### *Cytotoxicity assays*

The cytotoxic activity of TILs grown in IL-2 and/or GM-CSF was tested *in vitro* in a standard 4 h $^{51}$Cr release assay against fresh (cryopreserved) autologous tumour, one allogeneic tumour target (TU 59), K562, a NK-sensitive erythroleukaemia cell line, and Daudi cells, a NK-resistant lymphoma. A total of $5 \times 10^7$ target cells in a volume of 2 ml were labelled with 200 µCi of $^{51}$Cr (ICN Radiochemicals, Irvine, CA, USA) for 1 h at 37°C and washed three times before use. The $5 \times 10^3$ targets and the appropriate number of effectors at several effector–target (E/T) ratios (40:1, 20:1, 10:1 and 5:1) were plated in triplicate in a total of 0.2 ml of medium in 96-well round-bottom microtitre plates. After a 4 h incubation period the plates were centrifuged at 800 r.p.m. for 3 min and 100 µl of the supernatant was harvested, and counted on a gamma counter. The percentage specific lysis was determined as:

$$\frac{\text{Experimental counts} - \text{spontaneous counts}}{\text{Total counts} - \text{spontaneous counts}} \times 100$$

Target cells incubated in medium alone and with 1% sodium dodecyl sulphate were used to determine spontaneous and maximal release of chromium respectively. Cytotoxicity is expressed as lytic units (LU) per $1 \times 10^6$ cells. One lytic unit is defined as the number of effector cells needed to lyse 30% of $5 \times 10^3$ target cells.

### *GM-CSF receptor assays*

For the detection of cell-surface receptors specific for GM-CSF a previously published ligand-binding assay with $^{125}$I-labelled GM-CSF was used (DiPersio et al., 1988).

### *Statistical analysis*

The significance of differences in number of lytic units in assay and differences in percentages of positive cells of FACS analysis was determined using the Wilcoxon signed-rank test. A *P*-value < 0.05 was considered to indicate significance and two-tailed *P*-values were used.

### Results

### *Growth, expansion and proliferation*

The results of growth, maximal expansion and days in culture of TIL cultures under the various culture conditions are depicted in Table I. GM-CSF alone and when added to

**Table I** Growth, X-fold maximal expansion and maximal days in culture of tumour-infiltrating lymphocytes from RCC

| | Culture condition (IL-2 U $ml^{-1}$/GM-CSF nmol $ml^{-1}$) | | | | | | | | | | | |
|---|---|---|---|---|---|---|---|---|---|---|---|---|
| TIL | GM-CSF (500) | GM-CSF (1000) | IL-2 (8) | IL-2 + GM-CSF (8/1000) | IL-2 (40) | IL-2 + GM-CSF (40/1000) | IL-2 (400) Max. X-fold expansion | IL-2 (400) Max. days in culture | IL-2 + GM-CSF (400/500) Max. X-fold expansion | IL-2 + GM-CSF (400/500) Max. days in culture | IL-2 + GM-CSF (400/1000) Max. X-fold expansion | IL-2 + GM-CSF (400/1000) Max. days in culture |
| TIL 74 | NG | NG | NG | NG | NG | NG | 83.6 | 77 | 134.1 | 79 | 135.8 | 74 |
| TIL 75 | NG | NG | NG | NG | NG | NG | 69 | 65 | 87.4 | 60 | 91 | 62 |
| TIL 76 | NG | NG | NG | NG | NG | NG | 45.7 | 108 | 59.6 | 112 | 61.4 | 115 |
| TIL 77 | NG | NG | NG | NG | NG | NG | 62.4 | 127 | 77.9 | 118 | 78.4 | 112 |
| TIL 78 | NG | NG | NG | NG | NG | NG | 107.5 | 92 | 129.8 | 93 | 134.2 | 98 |
| TIL 80 | NG | NG | NG | NG | NG | NG | 21.6 | 69 | 28.6 | 59 | 27.1 | 65 |
| TIL 82 | NG | NG | NG | NG | NG | NG | 26.9 | 79 | 42.5 | 81 | 44.6 | 85 |
| Median | | | | | | | 62.4 | 79 | 77.9* | 81 | 78.4* | 85 |

*$P<0.02$ vs IL-2 control; NG, no growth.

suboptimal concentrations of IL-2 (8, 40 U $ml^{-1}$) failed to induce TIL growth in all cultures. Adding 500 nmol $ml^{-1}$ or 1000 nmol $ml^{-1}$ GM-CSF to the cell cultures together with a concentration of IL-2 which was alone sufficient to induce TIL growth (400 U $ml^{-1}$) resulted in the development of TILs. The expansion of TILs cultured in IL-2 + GM-CSF was significantly higher at both concentrations of GM-CSF when compared with TILs grown in IL-2 alone. The culture period of all TIL cultures did not differ regardless of the culture conditions.

The results of the [$^3$H]TdR incorporation assays can be seen in Table II. TILs incubated with IL-2 and GM-CSF at both concentrations studied proliferated significantly better than TILs grown in IL-2 alone.

### *Cytolytic activity*

Four hour $^{51}$Cr-release assays were performed in all seven TIL populations at an early stage of culture (days 25–45) and at later stage of culture (days 53–72). Fresh (cryopreserved) autologous and the allogeneic renal target cells (TU 59) were available for all experiments.

At an early stage of culture TILs grown with IL-2 and GM-CSF showed an enhanced killing activity against the autologous tumour target as well as against the allogeneic renal tumour target (Table III). The killing behaviour against the NK-cell sensitive K562 target and the NK-resistant Daudi cell target was unchanged when compared with the killing behaviour of corresponding TIL cultures activated with IL-2 alone. Killing of all TIL populations tested was always non-specific, as the allogeneic renal target, K562 and Daudi cells were lysed equally well or better independently of the culture condition. The same pattern in killing was observed at the second evaluation at a later stage of the cultures (data not shown), and killing remained also non-specific.

### *Phenotypical analysis*

Phenotypical analysis of TIL cultures (Table IV) supplemented with IL-2 or GM-CSF and IL-2 revealed similar percentages of cells positive for CD3 and CD3/CD56 respectively. TILs grown with 1000 nmol $ml^{-1}$ GM-CSF and IL-2 showed a significantly higher percentage of CD56$^+$ cells, while expression of CD56 was unchanged in TILs activated with 500 nmol $ml^{-1}$ GM-CSF + IL-2 when compared with TILs activated with IL-2 alone. The percentages of CD8$^+$ and CD4$^+$ cells were similar independent of the culture conditions. Expression of the IL-2 receptor (CD25) was unaffected by GM-CSF.

### *GM-CSF receptor analysis*

Two TIL populations were analysed for their ability to express the GM-CSF receptor. One population was activated with IL-2 400 U $ml^{-1}$ alone and the other with IL-2 400 U $ml^{-1}$ + GM-CSF 1000 nmol $ml^{-1}$. In neither of the

**Table II** Effects of GM-CSF on the proliferation of TILs from RCC

| | | Culture condition (U $ml^{-1}$ nmol $ml^{-1}$) | | |
|---|---|---|---|---|
| TIL | Culture (days) | IL-2 (400) | IL-2 + GM-CSF (400/500) | IL-2 + GM-CSF (400/1000) |
| TIL 74 | 22 | 27 120 | 27 681 | 37 917 |
| TIL 75 | 20 | 16 433 | 34 601 | 40 325 |
| TIL 76 | 20 | 14 233 | 21 990 | 21 213 |
| TIL 77 | 18 | 22 651 | 30 190 | 31 457 |
| TIL 78 | 23 | 15 385 | 27 341 | 30 501 |
| TIL 80 | 21 | 16 405 | 19 132 | 19 514 |
| TIL 82 | 18 | 17 291 | 23 477 | 23 537 |
| Median | | 16 433 | 27 341* | 30 501* |

[$^3$H]TdR (0.5 μCi per well) incorporation. Values represent the mean of c.p.m. in triplicate determinations. *$P<0.02$ vs IL-2 control.

**Table III** Cytolytic activity of TILs from RCC grown with IL-2 and GM-CSF

| | Lytic units against the following targets | | | | | | | | | | | |
|---|---|---|---|---|---|---|---|---|---|---|---|---|
| | Autologous tumour Culture condition ($U ml^{-1}/nmol ml^{-1}$) | | | Allogeneic renal tumour Culture condition ($U ml^{-1}/nmol ml^{-1}$) | | | K562 Culture condition ($U ml^{-1}/nmol ml^{-1}$) | | | Daudi Culture condition ($U ml^{-1}/nmol ml^{-1}$) | | |
| TIL | IL-2 (400) | IL-2 + GM-CSF (400/500) | IL-2 + GM-CSF (400/1000) | IL-2 (400) | IL-2 + GM-CSF (400/500) | IL-2 + GM-CSF (400/1000) | IL-2 (400) | IL-2 + GM-CSF (400/500) | IL-2 + GM-CSF (400/1000) | IL-2 (400) | IL-2 + GM-CSF (400/500) | IL-2 + GM-CSF (400/1000) |
| 74 | 6.5 | 10.5 | 10.0 | 1.8 | 2.8 | 2.1 | 24.0 | 25.0 | 27.0 | 19.5 | 25.7 | 34.0 |
| 75 | 6 | 7.6 | 7.4 | 2.9 | 4.1 | 5.3 | 40.5 | 32.4 | 65.4 | 37.0 | 44.4 | 69.0 |
| 76 | 0.4 | 1.9 | 2.4 | 9.2 | 7.5 | 8.5 | 22.8 | 48.3 | 50.5 | 11.6 | 14.0 | 14.3 |
| 77 | 3.2 | 1.8 | 3.8 | 0.3 | 18.0 | 16.2 | 11.9 | 11.7 | 11.7 | 21.6 | 17.9 | 15.7 |
| 78 | 8.2 | 10.6 | 9.8 | 9.2 | 7.8 | 9.8 | 13.9 | 8.9 | 9.1 | 15.0 | 14.3 | 9.8 |
| 80 | 1.7 | 12.7 | 13.5 | 1.3 | 4.0 | 4.1 | 23.3 | 20.5 | 26.9 | 6.2 | 8.4 | 8.7 |
| 82 | 0.8 | 3.5 | 3.9 | 5.3 | 7.4 | 7.9 | 7.0 | 12.1 | 20.4 | 3.5 | 7.5 | 7.4 |
| Median | 3.2 | 7.6* | 7.4* | 2.9 | 7.8* | 7.4* | 22.8 | 20.5 | 26.9 | 15.0 | 14.3 | 14.3 |

*$P < 0.05$ *vs* IL-2 control.

**Table IV** Phenotypical analysis of TILs from RCC grown in IL-2 and GM-CSF

| | | Median (range) positive cells per cent | | | | | | | | | | | |
|---|---|---|---|---|---|---|---|---|---|---|---|---|---|
| | | CD3 | | CD56 | | CD3/CD56 | | CD4 | | CD8 | | CD25 | |
| Culture condition ($U ml^{-1} nmol^{-1}$) | n | Days (25–45) | Days (53–72) | Days (25–45) | Days (53–72) | Days (25–45) | Days (53–72) | Days (25–45) | Days (53–72) | Days (25–45) | Days (53–72) | Days (25–45) | Days (53–72) |
| IL-2 (400) | 7 | 56 (20–97) | 38 (22–72) | 48 (2–76) | 44 (20–76) | 6 (1–13) | 7 (4–12) | 42 (11–74) | 42 (13–86) | 21 (9–3) | 22 (11–28) | 30 (4–83) | 29 (6–80) |
| IL-2 (400) + GM-CSF (500) | 7 | 51 (23–95) | 43 (23–72) | 40 (3–76) | 37 (22–72) | 7 (1–20) | 7 (4–18) | 33 (8–69) | 38 (11–76) | 27 (10–84) | 27 (15–56) | 24 (5–64) | 24 (6–48) |
| IL-2 (400) + GM-CSF (1000) | 7 | 67 (20–96) | 39 (20–79) | 72 (3–82)* | 73 (18–89)* | 8 (1–47) | 8 (5–19) | 23 (13–74) | 34 (12–76) | 21 (8–85) | 28 (8–72) | 36 (6–78) | 24 (7–42) |

*$P < 0.03$ *vs* IL-2 control.

two TIL populations tested could GM-CSF receptors be detected.

In summary, the addtion of GM-CSF to optimal concentrations of IL-2 resulted in a 25% increase in expansion, in a 66% increase in thymidine incorporation and in a 50% increase in CD56 expression of TILs from RCC. Moreover, there was a 138% increase in killing capacity of the tested IL-2/GM-CSF TILs against the autologous and allogeneic renal tumour targets, while the killing behaviour against the NK-sensitive cell line K562 and the NK-resistant cell line Daudi remained unchanged or tended to be lower.

### Discussion

GM-CSF exerts a wide array of biological activities on many cell types. Besides its stimulatory function on the proliferation of immature progenitors, it was soon recognised that GM-CSF could also enhance differentiated functions of mature effector cells (Lopez *et al.*, 1983; Vadas *et al.*, 1983; Weisbart *et al.*, 1985, 1986). Although still somewhat controversial, some well-designed *in vitro* studies have clearly demonstrated that the activity of GM-CSF is not restricted to monocytes/macrophages and granulocytes. Also, the proliferation and growth of T cells (Kupper *et al.*, 1987; Woods *et al.*, 1987; Santoli *et al.*, 1988; Herbelin *et al.*, 1989, 1990) as well as their *in vitro* killing behaviour (Masucci *et al.*, 1990) can be modulated by GM-CSF.

The first goal of our study was to investigate the ability of GM-CSF to induce TIL development from single-cell suspensions derived from RCC. GM-CSF at both concentrations (500 nmol $ml^{-1}$, 1000 nmol $ml^{-1}$) investigated failed to induce TIL development from RCC specimens when used as single activator. Also, when GM-CSF was added to tumour/lymphocyte suspensions together with suboptimal concentrations of IL-2 (8, 40 U $ml^{-1}$), no TIL development was observed. Only when the primary cell cultures were supplemented with GM-CSF and concentrations of IL-2 (400 U $ml^{-1}$) which sufficed alone for the activation and expansion of TILs was TIL development observed. Thus, GM-CSF cannot be assumed to be an independent growth factor for TILs derived from RCC, nor does GM-CSF facilitate the development of TILs when suboptimal doses of IL-2 are used.

However, TILs activated with GM-CSF and IL-2 differ significantly, in terms of proliferation, expansion and *in vitro* killing behaviour, from TILs activated with IL-2 alone. TILs grown with GM-CSF + IL-2 proliferated better than TILs activated with IL-2 alone, but the possible culture period was not affected. This enhancement of proliferation, coupled with similar time periods for which TILs could be maintained in culture, resulted in a significantly higher and more rapid expansion for TILs grown with either 500 nmol $ml^{-1}$ or 1000 nmol $ml^{-1}$ GM-CSF and IL-2. These results are in good agreement with the limited data available for peripheral T cells. Santoli *et al.* (1988) have demonstrated that GM-CSF enhances the short-term responsiveness of peripheral T cells to IL-2, and GM-CSF also potentiates the long-term growth of non-activated human lymphocytes and of lectin- and Ag-activated T cells in the presence of IL-2. Although in clinical investigations the number of activated cells reinfused to the patients and the effectiveness of treatment demonstrates no correlation thus far, most clinical protocols require the expansion of TIL cultures to at least $1 \times 10^9$ to $1 \times 10^{11}$ cells. Such cell counts are usually reached within 4–6 weeks of culture (Rosenberg *et al.*, 1988). Thus, this higher proliferation rate of TILs resulting in high cell counts when GM-CSF + IL-2 are used for activation would shorten the culture period, allowing an earlier onset of adoptive immunotherapy after surgical removal of the primary tumour.

Unlike TILs derived from melanoma (Itoh *et al.*, 1986; Muul *et al.*, 1987), the killing behaviour of TILs derived from RCC is non-specific in general, yet certain clones of RCC TILs have been isolated and exert autologous tumour-specific cytotoxicity (Koo *et al.*, 1991; Schendel *et al.*, 1993). TILs activated with GM-CSF and IL-2 showed a different killing behaviour *in vitro* when compared with the corresponding TIL cultures activated with IL-2 alone. The addition of GM-CSF to the culture medium resulted in enhanced killing of the autologous tumour target and the allogeneic renal tumour target. In contrast, lysis of the NK-sensitive K562 erythroleukaemia cell line and the NK-resistant Daudi lymphoma cell line remained unaffected. The percentages of $CD3^+$, $CD3^+/CD56^+$, $CD4^+$ and $CD8^+$ cells were similar in all TIL populations independent of the culture condition. The higher percentage of cells positive for the NK marker CD56 in TIL populations cultured with high concentrations of GM-CSF and IL-2 appears not to be responsible for the demonstrated enhanced killing, since TIL cultures supplemented with the lower concentration of GM-CSF also showed enhanced killing and a similar percentage of these cells were $CD56^+$ when compared with TILs activated with IL-2. Furthermore, the killing activity against the NK-sensitive K562 cell line remained unaffected independent of the concentration of GM-CSF used. The differences in the pattern of target lysis between TIL populations activated with IL-2 or GM-CSF + IL-2 were maintained over time in our experiments. Despite this enhancement of lytic activity of the autologous tumour target the killing behaviour of the tested TIL populations was always non-specific as the allogeneic targets were always lysed equally well or better. However, the fact remains that GM-CSF is able to enhance the lytic activity of RCC TILs against renal targets only. These data are in part similar to findings regarding the ability of GM-CSF to induce LAK-cell activity in peripheral lymphocytes. Masucci *et al.* (1990) demonstrated that peripheral lymphocytes activated with IL-2 and GM-CSF lysed Daudi cells and the human colorectal cancer cell line SW918 significantly better than IL-2-activated LAK cells. In that study, a 10-fold lower dose of IL-2 was required when GM-CSF was added as compared with IL-2 alone to generate a cytotoxic cell population with the same lytic activity. The authors assumed that GM-CSF might render more cells susceptible to IL-2 stimulation, since a higher cell fraction expressed CD25 when stimulated with IL-2 and GM-CSF. However, this is not reflected in our results, since the expression of CD25 in RCC TILs was similar whether or not GM-CSF was added to the medium.

The mode of action of GM-CSF's activity on lymphocytes in general and TILs in particular remains to be elucidated. Although the presence of a specific receptor might be assumed and the murine receptor specific for GM-CSF has been demonstrated in two cell lines of T-lymphocyte origin (Park *et al.*, 1986), the presence of the GM-CSF receptor on human lymphocytes has not been thoroughly investigated and has not as yet been demonstrated (Gasson, 1991). We were not able to demonstrate the presence of receptors specific for GM-CSF on the surface of mature RCC TILs. Although the expression of low numbers of the GM-CSF receptor on a minor subfraction of the TIL populations investigated might have been undetectable with the ligand-binding assay used, the pathway through which GM-CSF modulates T-cell actions, or at least TIL actions, appears to be an indirect rather than a direct one. After tumour processing the single-cell suspensions also contain macrophages and other mononuclear cells. Since GM-CSF has been shown to activate macrophages to enhance non-specific and specific immune responses against tumour cells, it might be speculated that one of these indirect effects could be the activation and stimulation of cells other than lymphocytes to release cytokines with T-cell-activating properties.

In summary, GM-CSF is not able to induce TIL development from RCC or to facilitate TIL activation induced by IL-2. However, TILs from RCC cultured with IL-2 and GM-CSF demonstrate significantly higher proliferation, resulting in a higher expansion of TIL cultures, and exert a higher killing activity against renal tumour targets *in vitro*. These findings provide a rational basis for the use of GM-CSF in the expansion of TILs, and further investigations are warranted. Clinical experience with systemic GM-CSF is more or less limited to the use of this cytokine to shorten chemotherapy-induced leucopenia. Unlike systemic IL-2 administration, GM-CSF application is rarely associated with serious side-effects (Morstyn *et al.*, 1988; Horn *et al.*, 1991; Steger *et al.*, 1992). Based on our results and the known properties of GM-CSF in improving host defence in immunocompromised patients by means of enhanced cytokine release and enhancement of cytolytic activity of neutrophils, eosinophils and macrophages (Weisbart, 1989), one might also speculate that GM-CSF could be of therapeutic value when administered systemically to patients receiving IL-2-based adoptive immunotherapy with TILs. The mode of action of GM-CSF in enhancing T-cell-mediated cytotoxic effects is not completely understood. Further *in vitro* and *in vivo* investigations with TILs and GM-CSF are needed to elucidate these issues.

**Acknowledgement**

GG Steger is a 1991 Max Kade Foundation grant recipient.

**References**

ALEXANDER J, RAYMAN P, EDINGER M, CONNELLY R, TUBBS R, BUKOWSKI R, PONTES E AND FINKE J. (1990). TIL from renal-cell carcinoma: restimulation with tumor influences proliferation and cytolytic activity. *Int. J. Cancer*, **45**, 119-124.

BEGLEY CG, NICOLA NA AND METCALF D. (1988). Proliferation of normal human promyelocytes and myelocytes after a single pulse stimulation by purified GM-CSF or G-CSF. *Blood*, **71**, 640.

BELLDEGRUN A, MUUL LM AND ROSENBERG SA. (1988). Interleukin-2 expanded tumor infiltrating lymphocytes in human renal cell cancer: isolation, characterization, and anti-tumor activity. *Cancer Res.*, **48**, 206-214.

CHAN JY, SLAMON DJ, NIMER SD, GOLDE DW AND GASSON JC. (1986). Regulation of expression of human granulocyte macrophage colony-stimulating factor. *Proc. Natl Acad. Sci. USA*, **83**, 8669.

DIPERSIO J, BILLING P, KAUFMAN S, EGHTESADY P, WILLIAMS RE AND GASSON JC. (1988). Characterization of the human granulocyte-macrophage colony-stimulating factor receptor. *J. Biol. Chem.*, **263**, 1834-1841.

DITONNO P, TSO CL, SAKATA T, DEKERNION JB AND BELLDEGRUN A. (1992). Regulatory effects of interleukin-7 on renal tumor infiltrating lymphocytes. *Urol. Res.*, **20**, 205-210.

DRANOFF G, JAFFEE E, LAZENBY A, GOLUMBEK P, LEVITSKY H, BROSE K, JACKSON V, HAMADA H, PARDOLL D AND MULLIGAN RC. (1993). Vaccination with irradiated tumor cells engineered to secrete murine granulocyte-macrophage colony-stimulating factor stimulates potent, specific, and long-lasting anti-tumor activity. *Proc. Natl Acad. Sci. USA*, **90**, 3539-3543.

FIBBE WE, KLUCK PMC, DUINKERKEN N, VOOGT PJ, WILLEMZE R AND FALKENBURG JHF. (1988). Factors influencing release of granulocyte-macrophage colony-stimulating factor from human mononuclear phagocytes. *Eur. J. Haematol.*, **41**, 352.

FIDLER IJ. (1985). Macrophages and metastasis - a biological approach to cancer therapy: presidential address. *Cancer Res.*, **45**, 4714-4726.

FIDLER IJ AND SCHROIT AJ. (1988). Recognition and destruction of neoplastic cells by activated macrophages: discrimination of altered self. *Biochim. Biophys. Acta*, **948**, 151-173.

FIEDLER W, SUCIU E, WITTLIEF C, OSTERTAG W AND HOSSFELD DK. (1990). Mechanisms of growth factor expression in acute myeloid leukemia (AML). *Leukemia*, **4**, 459.

FIGLIN RA, BELLDEGRUN A, MOLDAWER N, ZEFFREN J AND DEKERNION J. (1992). Concomitant administration of recombinant human interleukin-2 and recombinant interferon alpha-2a: an outpatient regimen in metastatic renal cell carcinoma. *J. Clin. Oncol.*, **10**, 414-421.

FINKE JH, TUBBS R, CONNELLY R, PONTES E AND MONTIE J. (1988). Tumor-infiltrating lymphocytes in patients with renal-cell carcinoma. *Ann. NY Acad. Sci.*, **532**, 387-394.

GASSON JC. (1991). Molecular physiology of granulocyte-macrophage colony-stimulating factor. *Blood*, **77**, 1131-1145.

HERBELIN A, MACHAVOINE F AND DY M. (1989). Effects of hematopoietic growth factor (GM-CSF: granulocyte-macrophage colony-stimulating factor) on thymocyte proliferation induced by interleukin-1 (IL-1). *Comp. Rendu. Acad. Sci. Paris*, **309**, 221-227.

HERBELIN A, MACHOVOINE F AND DY M. (1990). Potentiating effect of granulocyte-macrophage colony-stimulating factor on interleukin-1-induced thymocyte proliferation: evidence for an interleukin-2 and tumor necrosis factor-independent pathway. *Lymphokine Res.*, **9**, 155-165.

HERRMANN F, OSTER W, MEUER SC, LINDEMANN A AND MERTELSMANN RH. (1988). Interleukin-1 stimulates T lymphocytes to produce granulocyte-macrophage colony-stimulating factor. *J. Clin. Invest.*, **81**, 1415.

HORN TD, BURKE PJ, KARP JE AND HOOD AF. (1991). Intravenous administration of recombinant human granulocyte-macrophage colony-stimulating factor causes a cutaneous eruption. *Arch. Dermatol.*, **127**, 49-52.

ITOH K, TILDEN AB AND BALCH CM. (1986). Interleukin-2 activation of cytotoxic T lymphocytes infiltrating into human metastatic melanomas. *Cancer Res.*, **46**, 3011-3017.

KAUFMAN S, DIPERSIO DF AND GASSON JC. (1989). Effects of human GM-CSF on neutrophil degranulation *in vitro*. *Exp. Hematol.*, **17**, 800.

KAUSHANSKY K, LIN N AND ADAMSON JW. (1988). Interleukin 1 stimulates fibroblasts to synthesize granulocyte-macrophage colony-stimulating factors. *J. Clin. Invest.*, **81**, 92.

KOO AS, TSO CL, SHIMABUKURO T, PEYRET C, DEKERNION JB AND BELLDEGRUN A. (1991). Autologous tumor-specific cytotoxicity of human tumor-infiltrating lymphocytes derived from human renal cell carcinoma. *J. Immunother.*, **10**, 347.

KUPPER T, FLOOD P, COLEMAN D AND HOROWITZ M. (1987). Growth of an interleukin-2 interleukin 4-dependent T cell line induced by granulocyte-macrophage colony-stimulating factor (GM-CSF). *J. Immunol.*, **138**, 4288.

LEE TY, KOO AS, PEYRET C, SHIMABUKURO T, DEKERNION JB AND BELLDEGRUN A. (1991). The effects of interleukin-6 on tumor-infiltrating lymphocytes derived from human renal cell cancer. *J. Urol.*, **145**, 663-667.

LOPEZ AF, NICOLA NA, BURGESS AW, METCALF D, BATTYE FL, SEWELL WA AND VADAS M. (1983). Activation of granulocyte cytotoxic function by purified mouse colony-stimulating factors. *J. Immunol.*, **131**, 2983.

LOPEZ AF, WILLIAMSON J, GAMBLE JR, BEGLEY CG, HARLAN JM, KLEBANOFF SJ, WALTERSDORPH A, WONG G, CLARK SC AND VADAS MA. (1986). Recombinant human granulocyte-macrophage colony-stimulating factor stimulates *in vitro* mature neutrophil and eosinophil functions, surface receptor expression, and survival. *J. Clin. Invest.*, **78**, 1220.

MASUCCI G, RAGNHAMMER P, WERSALL P AND MELLSTEDT H. (1990). Granulocyte-macrophage colony-stimulating factor augments the interleukin-2-induced cytotoxic activity of human lymphocytes in the absence and presence of mouse or chimeric monoclonal antibodies (mAb 17-1A). *Cancer Immunol. Immunother.*, **31**, 231.

METCALF D, BEGLEY CG, JOHNSON GR, NICOLA NA, VADAS MA, LOPEZ AF, WILLIAMSON DJ, WONG GG, CLARK SC AND WANG EA. (1986). Biologic properties *in vitro* of a recombinant human granulocyte-macrophage colony-stimulating factor. *Blood*, **67**, 37.

MORSTYN G, LIESCHKE GJ, SHERIDAN W, LAYTON J, CEBON J AND FOX RM. (1988). Clinical experience with recombinant granulocyte colony-stimulating factor and granulocyte-macrophage colony-stimulating factor. *Semin. Hematol.*, **26**, 9-13.

MUUL LM, SPIESS PJ, DIRECTOR EP AND ROSENBERG SA. (1987). Identification of specific cytolytic immune response against autologous tumor in humans bearing malignant melanoma. *J. Immunol.*, **138**, 989-995.

OKAMURA S, HAYASHI S, ASANO Y, SHIBUYA T, OTSUKA T AND NIHO Y. (1988). Expression of the granulocyte macrophage colony-stimulating factor gene in leukemic blast cells from patients with acute non-lymphocytic leukemia. *Biomed. Pharmacother.*, **42**, 65.

PARK LS, FRIENFELD D, GILLIS S AND URDAL DL. (1986). Characterization of the cell surface receptor from human granulocyte-macrophage colony-stimulating factor. *J. Biol. Chem.*, **261**, 4177.

PLUZNIK DH, BICKEL M AND MERGENHAGEN SE. (1989). B lymphocyte derived hematopoietic growth factors. *Immunol. Invest.*, **18**, 103.

ROSENBERG SA, PACKARD BS, AEBERSOLD PM, SOLOMON D, TOPALIAN SL, TOY ST, SLION P, LOTZE MT, YANG JC, SEIPP CA, SIMPSON C, CARTER C, BOCK S, SCHWARTZENTRUBER D, WEI JP AND WHITE DE. (1988). Use of tumor-infiltrating lymphocytes and interleukin-2 in the immunotherapy of patients wtih metastatic melanoma. *N. Engl. J. Med.*, **319**, 1676–1680.

SANTOLI D, CLARK SC, KREIDER BL, MASLIN PA AND ROVERA G. (1988). Amplification of IL-2 driven T cell proliferation by recombinant human IL-3 and granulocyte-macrophage colony-stimulating factor. *J. Immunol.*, **141**, 519.

SCHENDEL DJ, GANSBACHER R, OBERNEDER R, KRIEGMAIR M, HOFSTETTER R, RIETHMÜLLER A AND SEGURADO O. (1993). Tumor-specific lysis of human renal cell carcinomas by tumor-infiltrating lymphocyte. *J. Immunol.*, **151**, 4209–4229.

SCHWARTZENTRUBER DJ, HOM SS, DADMARZ R, WHITE DE, YANELLI JR, STEINBERG SM, ROSENBERG SA AND TOPALIAN SL. (1994). *In vitro* predictors of therapeutic response in melanoma patients receiving tumor-infiltrating lymphocytes and interleukin-2. *J. Clin. Oncol.*, **12**, 1475–1483.

SIEFF CA, TSAI S AND FALLER DV. (1987). Interleukin 1 induces cultured human endothelial cell production of granulocyte–macrophage colony-stimulating factor. *J. Clin. Invest.*, **79**, 48.

SILBERSTEIN DS, OWEN WF, GASSON JC, DIPERSIO JF, GOLDE DW, BINA JC, SOBERMAN R, AUSTEN KF AND DAVID JR. (1986). Enhancement of human eosinophil cytotoxicity and leukotriene synthesis by biosynthetic (recombinant) granulocyte–macrophage colony-stimulating factor. *J. Immunol.*, **137**, 3290.

STEGER GG, LOCKER G, RAINER H, MADER RM, SIEDER AE, GNANT MFX, ABERER W AND JAKESZ R. (1992). Cutaneous reactions to GM-CSF in inflammatory breast cancer (letter). *N. Engl. J. Med.*, **327**, 286.

STEGER GG, PIERCE WC, FIGLIN R, CZERNIN J, KABOO R, DEKERNION JB, OKARMA T AND BELLDEGRUN A. (1994). Patterns of cytokine release of unselected and C28$^+$selected renal cell carcinoma tumor-infiltrating lymphocites (TIL). *Clin. Immunol. Immunopathol.*, **72**, 237–247.

THOMPSON JA, SHULMAN KL, BENYUNES MC, LINDGREN CG, COLLINS C, LANGE PH, BUSH WH, BENZ LA AND FEFER A. (1992). Prolonged continuous intravenous infusion interleukin-2 and lymphokine activated killer cell therapy for metastatic renal cell carcinoma. *J. Clin. Oncol.*, **10**, 960–968.

THORENS B, MERMOD JJ AND VASSALLI P. (1987). Phagocytosis and inflammation stimuli induce GM-CSF mRNA in macrophages through posttranscriptional regulation. *Cell*, **48**, 671.

TOPALIAN SL, SOLOMON D, ARUS FP, CHANG AE, FREERKSEN DL, LINEHAN WM, LOTZE MT, ROBERTSON CN, SEIPP CA, SIMON P, SIMPSON CG AND ROSENBERG SA. (1988). Immunotherapy of patients with advanced cancer using tumor-infiltrating lymphocytes and recombinant interleukin-2: a pilot study. *J. Clin. Oncol.*, **6**, 839–853.

TSO CL, DUCKETT T, DEKERNION JB AND BELLDEGRUN AS. (1992). Modulation of tumor-infiltrating lymphocytes derived from human renal cell carcinoma by interleukin-4. *J. Immunother.*, **12**, 82–89.

VADAS MA, NICOLA NA AND METCALF D. (1983). Activation of antibody-dependent cell-mediated cytotoxicity of the human neutrophils and eosinophils by separate colony-stimulating factors. *J. Immunol.*, **130**, 795.

WEISBERT RH. (1989). Colony stimulating factors and host defence. *Ann. Intern. Med.*, **110**, 297–303.

WEISBART RH, GOLDE DW, CLARK SC, WONG GG AND GASSON JC. (1985). Human granulocyte–macrophage colony-stimulating factor is a neutrophil activator. *Nature*, **314**, 361.

WEISBART RH, GOLDE DW AND GASSON JC. (1986). Biosynthetic human GM-CSF modulates the number and affinity of neutrophil f-Met-Leu-Phe receptors. *J. Immunol.*, **137**, 3584.

WEISS GR, MARGOLIN KA, ARONSON FR, SZNOL M, ATKINS MB, DUTCHER JP, GAYNOR ER, BOLDT DH, DOROSHOW JH AND BAR MH. (1992). A randomized phase II trial of continuous infusion interleukin-2 or bolus injection interleukin-2 plus lymphokine activated killer cell for advanced renal cell cancer. *J. Clin. Oncol.*, **10**, 275–281.

WOODS A, WEST J, RASMUSSEN R AND BOTTOMLY K. (1987). Granulocyte-macrophage colony-stimulating factor produced by cloned L3T4a + class II-restricted T cells induce HT-2 cells to proliferate. *Immunology*, **138**, 4293–4297.

ZINZAR SN, SVET-MOLDARSKY GJ, FOGH J, MANN PE, ARLIN Z, ILIESCU K AND HOLLAND JF. (1985). Elaboration of granulocyte–macrophage colony-stimulating factor by human tumor cell lines and normal urothelium. *Exp. Hematol.*, **13**, 574.